# Lightweight Mortar Incorporating Expanded Perlite, Vermiculite, and Aerogel: A Study on the Thermal Behavior

**DOI:** 10.3390/ma17030711

**Published:** 2024-02-02

**Authors:** José Balbuena, Mercedes Sánchez, Luis Sánchez, Manuel Cruz-Yusta

**Affiliations:** 1 Fundación CIAC, C/Astronomo Kepler 4.2, P.T. Rabanales, 21, E-14014 Córdoba, Spain; donjosebj@gmail.com; 2 Departamento de Química Inorgánica, Instituto de Química para la Energía y Medioambiente, Universidad de Córdoba, Campus de Rabanales, E-14014 Córdoba, Spain; msmoreno@uco.es (M.S.); luis-sanchez@uco.es (L.S.)

**Keywords:** thermal conductivity, mortar, lightweight aggregate

## Abstract

Climate change is compelling countries to alter their construction and urbanization policies to minimize their impact on the environment. The European Union has set a goal to reduce greenhouse gas emissions by 55%, recognizing that 50% of its emissions originate from maintaining thermal comfort within buildings. As a response, the EU has developed comprehensive legislation on energy efficiency. In this article, special mortars using aerogel, perlite, and vermiculite as lightweight aggregates were prepared and studied to enhance the thermal properties of the mortar. Their thermal properties were examined and, using a solar simulator for both hot and cold conditions, it was found that varying proportions of these lightweight aggregates resulted in a mortar that provided insulation from the exterior up to 7 °C more than the reference mortar in warm conditions and up to 4.5 °C in cold conditions.

## 1. Introduction

The climatic change occurring in recent decades is dealing with increasing adverse environmental conditions in the cities, even harming the quality of life and the health of the citizens. The energy demand of buildings to maintain comfortable conditions is becoming more and more pronounced.

The European Union, in its Directive 2018/84 [1], commits to “*stablishing a sustainable, competitive, secure and decarbonised energy system. The Energy Union and the Energy and Climate Policy Framework for 2030 establish ambitious Union commitments to reduce greenhouse gas emissions further by at least 40% by 2030 as compared with 1990, to increase the proportion of renewable energy consumed, to make energy savings in accordance with Union level ambitions, and to improve Europe’s energy security, competitiveness and sustainability*”.

In this same directive, the proposal is to establish a sustainable, competitive, and decarbonized energy system by 2050. To achieve this goal, Member States need measures aimed at reaching the long-term greenhouse gas emissions target and decarbonizing the building stock, which will be responsible for approximately 36% of all CO_2_ emissions in the Union by 2050.

On the other hand, Directive 2023/1791 [2] is even more demanding and proposes to increase the Union’s climate ambition for 2030 by raising the target for reducing greenhouse gas emissions to at least 55% below 1990 levels. This directive also highlights that the potential for energy saving is significant and sets a challenge regarding the building stock, as 75% of buildings exhibit low energy performance.

The 2015 Paris Agreement on climate change, known as the “Conference of the Parties to the United Nations Framework Convention on Climate Change (COP 21),” [3] drives the Union’s efforts to decarbonize its building stock. Considering that nearly 50% of the Union’s final energy consumption is allocated to heating and cooling, with 80% of this occurring in buildings, achieving the Union’s energy and climate change objectives is tied to its efforts to renew its building stock by prioritizing energy efficiency, applying the “efficiency first” principle, and exploring the deployment of renewable energy sources.

To achieve these objectives, a concept known as nZEB (nearly Zero Energy Buildings) has been introduced. These are buildings with a very high energy performance and at least 30% of the consumed energy must be covered from renewable sources [4]. To accomplish this, it is necessary to minimize the energy consumption of the building.

Minimizing the energy consumption of a building involves implementing various strategies and technologies to improve its energy efficiency. These strategies range from using efficient LED-based lighting systems or low-emissive windows with airtight sealing to employing bioclimatic designs that leverage climatic conditions for the benefit of the building. One of these options is to use more efficient insulation by enhancing the insulation of walls and roofs to minimize heat transfer between the interior and exterior of the building. This, in turn, reduces the need for heating and cooling systems, thereby decreasing energy consumption.

An energy-efficient building must be designed to be insulated from the cold, heat, wind, and/or rain outside, i.e., keep thermal comfort conditions regardless of the weather outside.

The development of eco-energy-efficient thermal materials is necessary to mitigate the cooling needs of buildings [5]. In addition, these materials can be applied to the design of new buildings and, above all, to the renovation of existing dwellings. This is one of the challenges facing society today, to minimise energy losses in buildings and achieve more sustainable cities [6].

Numerous authors highlight that the major energy loss in buildings occurs through the walls or the roof. For instance, Almujahid and colleagues [7] found that over a third of the building’s energy was lost due to poor insulation conditions in the walls. Kossecka and colleagues [8] examined the performance of an insulation layer on the wall in six different climates in the US and demonstrated that using a thermal insulation material on walls improved the thermal efficiency of buildings. These studies have even extended to differentiate between the exterior walls, i.e., the building envelope, and the interior walls, i.e., partitions [9]. 

Based on the above data, it seems logical that one of the most effective ways to avoid energy losses through walls is to work on the thermal insulation of the building envelope. The use of insulating materials in the building envelope will reduce the energy consumption needed to maintain indoor comfort and reduce the environmental impact of buildings by reducing greenhouse gas emissions [10]. 

Within these new materials, rendering mortars are a building’s first barrier against external climatic factors. Therefore, an improvement of the thermal insulation properties in these materials involves a significant reduction in the building’s energy consumption [11]. 

The use of lightweight aggregates in the composition of insulating mortars and concretes has significant potential for improving the thermal performance of the cementitious material and, thus, reducing thermal losses. Lightweight aggregates can be derived from natural compounds, such as expanded perlite or vermiculite [12,13], waste, such as end-of-life tires or industrial waste [14,15], and other industrial products, such as aerogel or expanded polystyrene (EPS) [16].

A deeper analysis of the use of these lightweight aggregates shows that perlite and vermiculite are widely used in various construction elements such as bricks, mortars, pipes, and soils, with different objectives such as thermal insulation, fire resistance, sound insulation, drainage ease, etc. Among them, their use for thermal insulation is the most widespread [17,18], achieving improvements that even reach up to an 83% reduction in thermal conductivity. The particle size of lightweight aggregates is a critical parameter in the achieved thermal conductivity values [19]. Gandage and col. [20] used different percentages of perlite in the mortar composition and measured thermal conductivity at various temperatures. They found that as the temperature increased, the conductivity decreased for the same substitution percentage. Similar results have been described by Malek and col. [21]. They observed a gradual reduction in thermal conductivity from 1.9 Wm^−1^K^−1^, when the mortar had no perlite, to 0.69 Wm^−1^K^−1^, when the perlite replacement reached 100%, which resulted in a 62% reduction in thermal conductivity.

Both perlite and vermiculite have also been studied in combination with other isolating materials, such as the mixture of vermiculite and PCMs studied by Chung and col. [22]. In this case, they found a 15% reduction in thermal conductivity when using perlite and a 40% reduction when using vermiculite. However, there is a gap in the literature about cement-based lightweight mortars produced with combinations of them. 

On the other hand, aerogel is a much less studied material as its development occurred in the last 10 years. Nevertheless, numerous works have been conducted with the aim of improving building insulation [23,24,25,26].

In this work, two novelties are presented. Firstly, the assessment of the synergic effect of the combination of perlite, vermiculite, and aerogel on the thermal response of cementitious mortars. For this purpose, the physicochemical characterization of both the raw materials and the prepared mortars is determined. Furthermore, a novel test is proposed for simulating the exposure of insulating materials to real conditions of continuous heating or cooling using a hand-made system. The mechanical performance of the developed insulation mortars is also evaluated to ensure that they meet the minimum requirements for being applied as rendering mortars according to standard EN 998-1:2018 [27]. 

## 2. Materials and Methods

### 2.1. Mortar Components and Materials

Low thermal conductivity mortars were produced incorporating three different materials commonly used as thermal insulation materials (aerogel, perlite, and vermiculite) in a Portland cement-based mixture. Different types of mixtures were defined to obtain optimal thermal conductivity properties in each case. Thus, the mixture composition was selected based on its thermal conductivity and heat transfer response, and by assuring an acceptable compressive strength. Once the optimum mixtures were selected, test specimens were prepared to evaluate their thermal and mechanical properties.

The different components used in the preparation of the mortar mix are described in this section.

#### 2.1.1. Portland Cement

All samples were prepared with the same amount of BL I 52.5 R white cement.

#### 2.1.2. Aggregates

Two different types of aggregates were used. On the one hand, dolomitic sand with a selected grain size between 0.01 and 1.0 mm was used. On the other hand, a calcareous filler with a much smaller grain size between 0.001 and 0.1 mm was also used. Both were supplied by Cemkosa (Córdoba, Spain), a local mortar company.

#### 2.1.3. Additives

All samples were prepared with the same commercial additives that were added in equal amounts. The additives used were:

- Polypropylene fibre between 7 and 14 mm in length, with a specific gravity of 0.89 g cm^−3^, a modulus of elasticity of 3.5 kN mm^−2^, and a tensile strength between 0.55 and 0.79 kN mm^−2^.
- Absorbent SIKAMOR-A, supplied by SIKA, to adjust water retention and workability.
- Water-repellent SIKAGUARD 917, supplied by SIKA, to prevent the water-attracting effect of the thermal insulation materials in the mortar formulation.

#### 2.1.4. Thermal Insulation Materials

- Perlite is a vitreous volcanic rock that contains 4% water. Expanded perlite is an extremely light and white granular material. Perlite was supplied by MERCOR TECRESA (Madrid, Spain), a national mortar manufacturer. Its chemical composition is 61.61% SiO_2_, 8.84% Al_2_O_3_, 16.92% Fe_2_O_3_, 1.8% TiO_2_, 4.11% K_2_O, 6.02% MgO, and 0.7% others. It has a density of 35–65 Kgm^−3^ and a specific heat of 0.84 KJKg^−1^K^−1^.
- Vermiculite is the name of a group of laminar hydrated minerals composed of aluminium, iron, and magnesium silicates, and is like mica in appearance. It is a safe and light-coloured inert material whose chemical formula is (Mg,Fe^2+^,Fe^3+^)_3_[(Al,Si)_4_O_10_](OH)_2_·4H_2_O. Vermiculite was supplied by MERCOR TECRESA, a national mortar manufacturer. Its chemical composition is 77.47% SiO_2_, 10.84% Al_2_O_3_, 1.5% Fe_2_O_3_, 1.7% TiO_2_, 6.27% K_2_O, 1.4% MgO, and 0.81% others. It has a density of 50–120 Kgm^−3^ and a specific heat of 0.84–1.08 KJKg^−1^K^−1^.
- Aerogels are a colloidal material, like a gel. In its preparation the liquid phase is replaced by a gas, resulting in a very porous solid with a very low density. The aerogel used in this study was an amorphous silica gel with up to 98% air inside. The aerogel was supplied by the company Green Earth Aerogel Technologies. Its chemical composition is > 97% SiO_2_ with a density of 156 Kgm^−3^ and a specific heat of 0.99 KJKg^−1^K^−1^.

Scanning electron microscopy images were used to understand the morphology of these lightweight aggregates.

Eight mortar formulations were used to study the influence of these insulating additives on the thermal properties of the mortar. The additions were estimated to simulate, as closely as possible, the actual formulation of a thermal insulating mortar (see Table 1). The nomenclature used to name the samples is R for the reference, P for the samples that contained perlite, V for the samples that contained vermiculite, and A for the samples that contained aerogel. The numbers after the letter refer to the percentage of substitution of dolomitic sand with the thermal insulation material. In the case of samples with two different substitutions, only two letters appear, referring to the substituted material in its maximum percentage.

Mortars with eight different compositions were prepared by varying the content of dolomitic sand and thermal insulation materials. The dolomitic aggregate, one of the main components of the mortar, is the one that most contributes to the mortar’s thermal conductivity. As can be seen in Table 2, the dolomitic aggregate is the raw material with the highest thermal conductivity. On the other hand, thermal insulator materials have the lowest thermal conductivity (see Table 2). Based on this aspect, and to improve the thermal behaviour, dolomitic sand was partially replaced by materials with lower conductivity.

### 2.2. Mixing, Moulding and Compaction

Tap water was used for preparing the mortar samples. The percentage of water in each mix varied from 19.9% to 75.4%. The water-cement ratio was adjusted to obtain a minimum horizontal flow of 160 mm.

The samples were mechanically mixed in an automatic mixer according to the recommendations of the standard EN 196-1:2018 [28].

The mixing procedure for fresh mortar mixtures was followed. Firstly, the additives and water were mixed. Next, cement, sand, and water were added to the mixer and mixed for three minutes. Then, the filler and lightweight aggregate were added and mixed for 1.5 min. Finally, polypropylene fibres were added, dispersed to avoid the formation of agglomerates, and mixed for another three minutes. The mixing vessel was then removed and checked to ensure that no unmixed residues remained on the walls of the vessel.

The thermal insulating materials (perlite, vermiculite, and aerogel) have a high water absorption capacity. For this reason, more water was added to these mortars to achieve the desired workability degree.

Immediately after the mixing process, the cementitious mix was cast in two layers. Each layer was compressed on a compacting table by performing 40 shocks. In this work, two different moulds were used: 4 cm× 4 cm× 16 cm to test the mechanical properties and 16 cm× 12 cm × 1.5 cm to test the thermal properties in real conditions. Six specimens for each series were cast to perform both the mechanical and thermal tests. Fresh properties, density, and consistency were determined immediately after the mixing. The UNE-EN 1012-2:1996 standard [29] was used for determining the horizontal flow and the density was measured according to the UNE-EN 1015-6:1999 standard [30].

### 2.3. Curing and Conditioning

After compacting, all moulds were kept in plastic bags in laboratory conditions (relative humidity of 95 ± 5% and 20 ± 2 °C) for 24 ± 1 h to prevent fast evaporation of water from the specimens. Then, the specimens were demoulded and stored according to the UNE-EN 1015-11:2020 standard [31] until testing.

### 2.4. Thermal Performance

The characterization of the thermal properties of the mortars was carried out with a commercial system DECAGON brand model KD2Pro. This equipment is based on the thermal needle probe procedure (TNP). It involved inserting a needle into the material, heating the needle with an electric current for a period, and using a thermocouple to measure the temperature variation over time. Subsequently, through a series of mathematical calculations based on finite elements, the thermal conductivity value was obtained. This equipment is portable and, depending on the probe, allows us to measure the Thermal Conductivity, Thermal Resistivity, Specific Heat, and Thermal Diffusivity of the material. The measurement principle is based on the method of “Transient Line Heat Source”. Measures were recorded in one-second intervals during a 90 s warm-up and cool-down cycle. This equipment met the specifications of the IEEE 442-1981 [32] and ASTM D5334-08 [33] standards. The measurement was carried out in two different ways. For the raw material, a cylindrical pot, 5 cm in diameter, was filled with the powdered raw materials and the probe was inserted in the middle. For mortars, test prims of 4 × 4 × 8 cm were prepared and the middle of the 4 × 4 face was drilled to insert the probe. To maximize the contact with the material, the probe was coated with a conductive paste.

**Figure 1:**
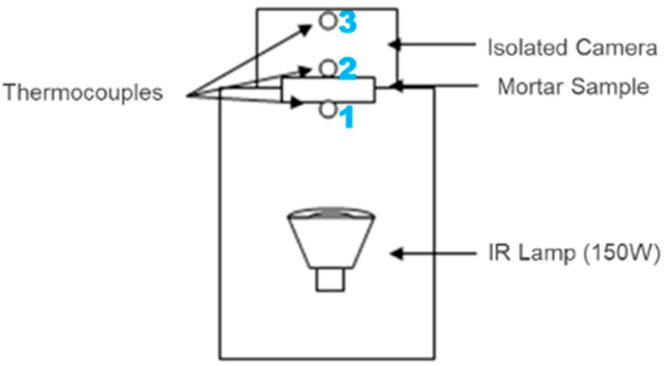
Schematics homemade device to evaluate the thermal behaviour in real conditions.

A homemade device was made to evaluate the thermal performance in exposure conditions that simulated a real situation. The cross-section schematic diagram of the device is included in Figure 1. As can be seen, there are three thermocouples in the device. he first one (1) is located between the lamp used for heating and the sample. The second (2) is placed on the other face of the sample to evaluate the difference in the temperature between both faces of the sample. Finally, a third thermocouple (3) is placed 13 cm apart from the non-exposed face, but within an isolated space of about 13 cm^3^, which allows us to see the temperature evolution. 

**Figure 2:**
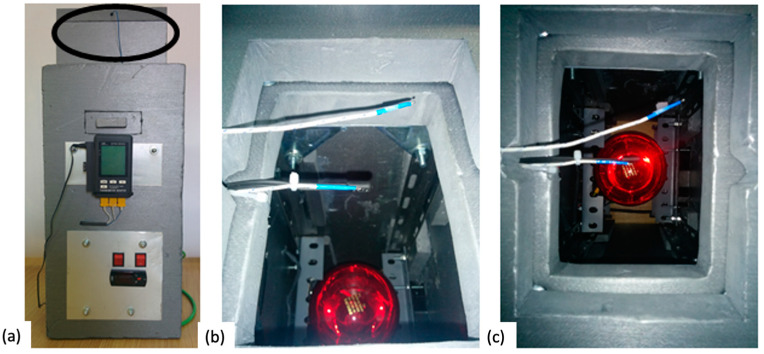
Homemade simulator: (**a**) general view, and (**b**) and (**c**) thermopar detail.

The manufacturing of mortar pieces using a specific mould of 160 × 120 × 15 mm, designed to create prismatic specimens, established a uniform standard for the samples. Precision in the dimensions of the specimens is crucial to ensure consistent and comparable results in experiments involving mortar. By placing each mortar sample between thermocouples (1) and (2), a thermal measurement system was established, enabling the accurate monitoring of temperature changes through the specimens. The mortar was located within a chamber of extruded polystyrene (XPS) which plays a crucial role, as shown in Figure 2. The XPS acts as an insulating material, aiding in maintaining a constant temperature around the samples and minimizing heat losses to the surroundings, promoting the reproducibility of results.

The sample is irradiated with an IR lamp for heating the sample from the side where the thermocouple (1) is placed.

In Figure 2a, a photo of the homemade simulator can be observed. The measurement was carried out by simulating two different ambient conditions: severe hot and severe cold. For both situations, the same simulator was used, but for severe cold conditions, the simulator was located inside a freezer. Other authors have also used a similar homemade set-up for analysing the thermal performance of mortar [34].

The test for simulating extreme heat temperature was carried out by placing the entire system in a room at room temperature and setting the temperature of the heating source to 40 °C. The test continued until thermocouple 2 reached a stable temperature for at least 1.5 h. The test for simulating extreme cold temperature was carried out similarly, with the difference being that the entire system was placed in a room with a controlled temperature fixed at 4 °C. For cold tests, the upper part of the system (see mark in Figure 2a) that holds thermocouple 3 was removed, leaving it exposed to the room environment to guarantee that the external temperature was constant during the test. In cold conditions, the sample was heated in the bottom part of the equipment during the test while the other part of the sample was exposed to cold conditions. The idea is to simulate a situation of exposure to a cold environment on the exterior (winter) and heating indoors. The heating of the sample promoted an increase in the temperature with time, and what was being evaluated was the loss of heat due to external cold conditions. Thus, in this case, the aim was to achieve the lowest possible temperature on the surface exposed to the external environment related to the lowest loss of heat.

## 3. Results and Discussion

### 3.1. Fresh Paste and Early-Age (7-Days Curing)

#### 3.1.1. Density and Consistency

To evaluate the consistency of the mixes a flow table was used. In Table 3, the average of four measurements per sample is shown. The consistency values in all cases ranged from 160 to 185 mm.

Concerning the water amount, the addition of thermal insulation materials implies the need to increase the water amount. In some cases, even four times more water was needed. The greater the volume occupied by the lightweight aggregate, as in the case of mixtures with two different substitutions, the greater the amount of water required [35].

In the mortar incorporating perlite as a thermal insulation material, there was a direct relationship between increasing the percentage of perlite and the amount of water required to keep the workability at the same value. The same behaviour was observed when vermiculite was used as the thermal insulation material, as a higher water demand was registered with the increasing content of vermiculite, maintaining a similar consistency to the samples. However, when aerogel was used, lower water content was needed compared to the sample with the same content of perlite (P10), and it had a similar content to the sample with an even lower content of vermiculite (V7). This discrepancy is likely due to the aerogel’s potential to accelerate the setting time, leading to lower water requirements during the mortar preparation. This behaviour was consistently observed during the mortar mixture. In the case of mortars with two different lightweight materials, the total substitution of dolomite was higher, and the water demand increased considerably, especially if the mortar contained vermiculite in its composition, to attain comparable workability values as the other mixtures. These observations underscore the importance of carefully calibrating water proportions to maintain desired workability when different thermal insulation materials are considered.

The visual analysis of the different mortar mixes saw that perlite stood out as the lightest, which occupied a volume three times greater than that of vermiculite and an impressive fifty times higher volume compared to dolomitic sand. 

This suggests that the binary mixture may have a propensity to absorb water more readily, requiring careful adjustment of the water content during the preparation of the mortar.

With all this, it can be deduced that the higher dosage of thermal insulation materials results in less-plastic mortars that need higher water content to reach the appropriate workability.

The analysis of the dry powder density data, included in Table 3, revealed that, for any of the mortars incorporating lightweight materials, a significant decrease in the mix density was obtained when substitutions above 10% of the dolomite sand were considered. The highest reduction was observed for the mixtures with perlite in their composition which exhibited the lowest density. This finding suggests that perlite is the lightest among the three thermal insulation materials analyzed in this study, even lighter than aerogel.

As anticipated, upon inspecting the density data in its fresh state after the first 24 h curing (see Table 3), the samples with perlite in their composition proved to be the heaviest, signifying a greater capacity for water absorption. Conversely, the aerogel displays the opposite behaviour, retaining less amount of water than perlite. 

Upon carefully examining this trend, and meticulously considering the data collected in Table 3 which details the consistency and water content during the cement mixing process, it can be concluded that the aerogel exhibits a behaviour that tends to reduce the workability of the material. Other authors attribute this behaviour to the irregular morphology of the aggregate particles present in the aerogel [36,37], also confirmed by SEM-analysis in the present study, as shown in the next section.

#### 3.1.2. Flexural and Compressive Strength after 7-Days Curing

The data for flexural and compressive strength can be seen in Table 4. When analyzing the flexural strength data, it could be observed that significant strength losses were registered for almost all the samples, with the exception of the sample labelled P3 which showed a strength loss of 5.7%. The rest of the samples exhibited a much higher loss of strength and even exceeded 90% in the case of the sample with two different lightweight aggregates, labelled AV. Sample A showed a slightly lower loss of strength compared to the others, which could be attributed to the previously mentioned irregular morphology.

On the other hand, the compressive strength data showed a similar tendency to the described for flexural strength. The reference (R) and P3 samples presented similar strength values, but these values were much worse for the rest of the samples. In the case of the compressive strength, the losses registered were above 60% in all cases, reaching up to a 95% loss of strength in the case of the sample AV, with a total substitution of 25% of the dolomite aggregate. However, these low strength values are acceptable for thermal mortars [38] where compressive strengths higher than 0.4 N/mm^2^ are required at 28 days according to the EN 998-1:2018 standard [27].

**Figure 3:**
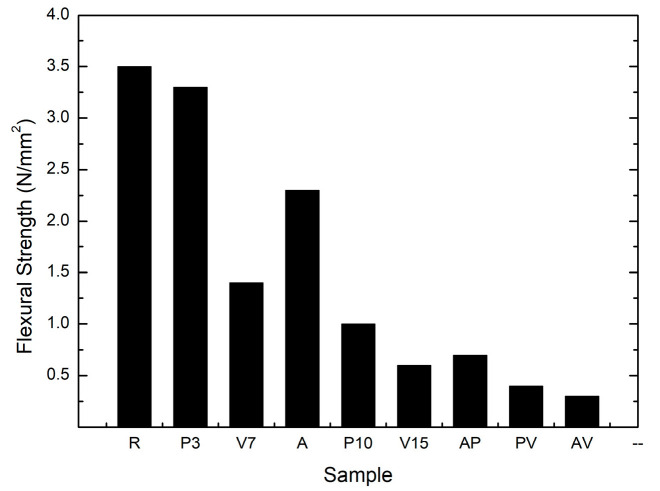
Flexural strength data for mortars with different mixtures of lightweight aggregates.

In Figure 3 the flexural strength for the different lightweight materials is plotted. A logical relationship could be observed, as lower values of mechanical resistances were registered. Nevertheless, it should be noted that samples that contained aerogel as a lightweight aggregate exhibited a higher resistance than would be expected for their percentage of addition (samples A and AP). These results are in line with those reported by other researchers in previous studies [39,40].

**Figure 4:**
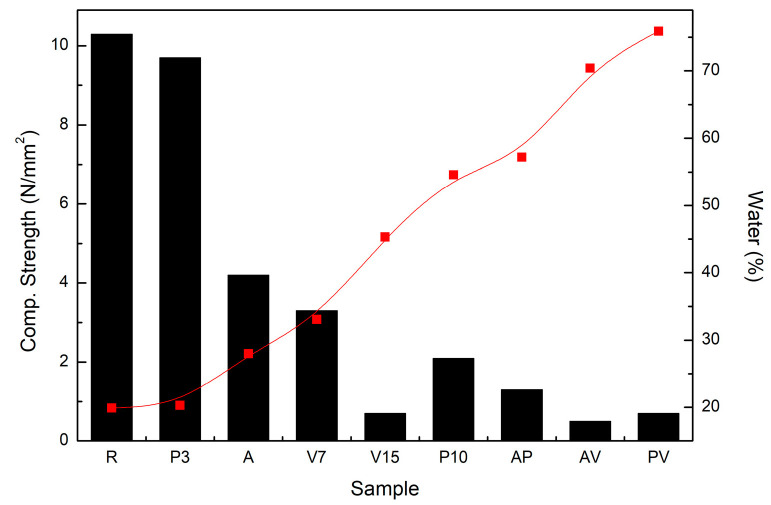
Correlation between compressive strength and the amount of mixing water used for each sample.

A direct correlation was observed between the amount of mixing water used for the mortar preparation and the compressive strength of the mortars after 7 days of curing (Figure 4). As can be seen, as the amount of mixing water used increased, the mechanical strength of the material decreased. This is due to the increased porosity expected in samples with a higher amount of mixing water. If the mortars in the graph are arranged in ascending order of mixing water, it can be observed that the mortars that require the most mixing water are generally those that include a substitution of 15% vermiculite in their formulation. Following the same reasoning, from the same graph, it is possible to deduce that mortars with 15% vermiculite in their formulation exhibited the lowest mechanical strengths.

### 3.2. Hardened State Mortar

#### 3.2.1. Characterization after 28 Days of Curing

In Table 5, the data on mechanical resistances and density after 28 days of curing are shown. Regarding the density data, it can be observed that, in general, all decreased compared to the data collected in Table 3, corresponding to early ages (7 days), which can be explained due to the advance in the formation of hydrated solid phases. The densities decreased so much that they were even comparable to powder densities. However, it should be noted that in the case of mortars with a lightweight aggregate type, the densities in the hardened state were lower than those collected in powder. For mortars with two lightweight aggregate types, the values of powder density were not reached. With the advance of the curing age, the porosity of the sample was already fully defined, and a lower percentage of water remained within the pores.

A deeper analysis of the data indicated that only the sample labelled P3 maintained similar values to the reference formulation, which was significantly higher than the values recorded for the other mortars. This can be due, on one hand, to the small percentage of substitution in this mix and, on the other hand, to the fact that the size of the perlite was smaller than that of the other lightweight aggregates, thus, trapping less air inside. For samples with only one type of lightweight aggregate, a direct relationship between the decrease in density values and the increase in substitution was found, with the lowest values measured for the V15 and A samples. When simultaneous substitutions of two lightweight aggregates were considered, the density values decreased significantly, reaching even 700 kg/m^3^, which was related to a higher percentage of total substitution.

As can be seen in Table 5, a relationship between density values and mechanical strengths (flexural and compressive) was deduced, with the lightest samples being the least resistant and the heaviest ones the most resistant. The formulation with more amounts of lightweight aggregate had the worst mechanical behaviour, and also the lowest density values.

Only the P3 mortar sample had compressive strength values similar to the values presented by the reference mortar. This could be related not only to the lesser replacement of dolomite aggregates in this sample but also to the fact that the chemical composition and the size of perlite were like that of the aggregate it replaced. 

The mortar with vermiculite, even in a low proportion, did make the values of compressive strength drop by half, and if the increase of the percentages added in the dosage is considered, this value dropped even more. This may be because an expanded vermiculite was being used as aggregate, and it is possible that it exfoliates, resulting in a loss of mechanical properties, especially in relation to flexural strength. 

In the case of hybrid replacement with two different lightweight aggregates, the one with the best mechanical behaviour was the mixture that contained aerogel and perlite, probably because the small size of the perlite caused a binding effect on the aerogel giving it better mechanical properties.

Compared to the reference mortar, after 28 days of curing, all lightweight mortars, except P3, showed significant mechanical strength losses: over 75% for flexural strength and 80% for compressive strength. Despite these poor mechanical strength values, it should be noted that for thermal mortars, the compressive resistance values collected in Table 5 are in the range required for category CSI–CSII lining mortars according to the EN 998-1:2018 standard [27].

**Figure 5:**
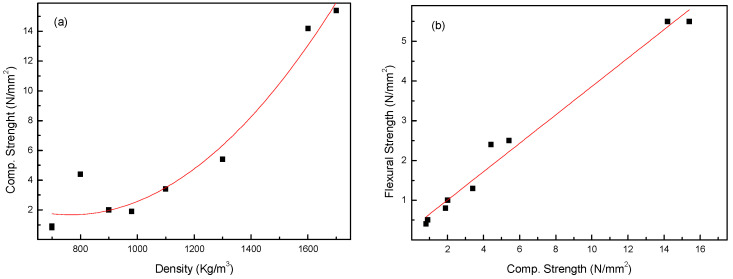
Correlation between compressive strength and density (**a**) and between flexural tensile strength and compressive strength (**b**) for lightweight mortar after 28 days of curing.

Considering the correlation between density and compressive strength (Figure 5a), it could be observed as an acceptable fitting to a second-order polynomial equation, with R^2^ = 0.82. Between the flexural and compressive strengths (Figure 5b), a linear correlation with R^2^ = 0.97 was obtained. These values are indicative that the findings are coherent.

#### 3.2.2. Thermal Conductivity

In Table 6, the thermal properties of the different mortars are detailed. Sample P3 presented high thermal conductivity values, close to the reference mortar, probably due to the small amount of lightweight aggregate in the mix. Due to this bad performance, this sample was discarded for the thermal test.

It can be highlighted that the best performance in terms of thermal conductivity properties was obtained for the sample that contained aerogel as the only lightweight aggregate. In this case, the decrease in thermal conductivity was 75% compared to the thermal conductivity of the reference mortar. This good behaviour may be due to the small size of the aerogel particles. These particles, being very small, increase the contact surface, causing a decrease in thermal conductivity.

It can also be observed that the samples that contained perlite as a lightweight aggregate either did not improve the thermal conductivity of the reference mortar or the improvement did not reach 50%. These results are similar to those found by other researchers [41]. This may be due to the small percentage of addition (sample P3) [18] or because this lightweight aggregate requires a greater amount of water that remains trapped in the internal pores of the perlite [42].

In general, for mortars in which a mix of lightweight aggregates was used (samples AP, AV, and PV), the conductivity values were lower than those obtained when only one type of lightweight aggregate was used. This is probably because, in the case of blends of lightweight aggregates, the percentage of substitution was higher. 

It is interesting to observe that when mixing aerogel with another type of lightweight aggregate (perlite or vermiculite), the resulting mortar slightly worsened its thermal behaviour, resulting in higher thermal conductivity compared to the mortar using only aerogel as the lightweight aggregate.

**Figure 6:**
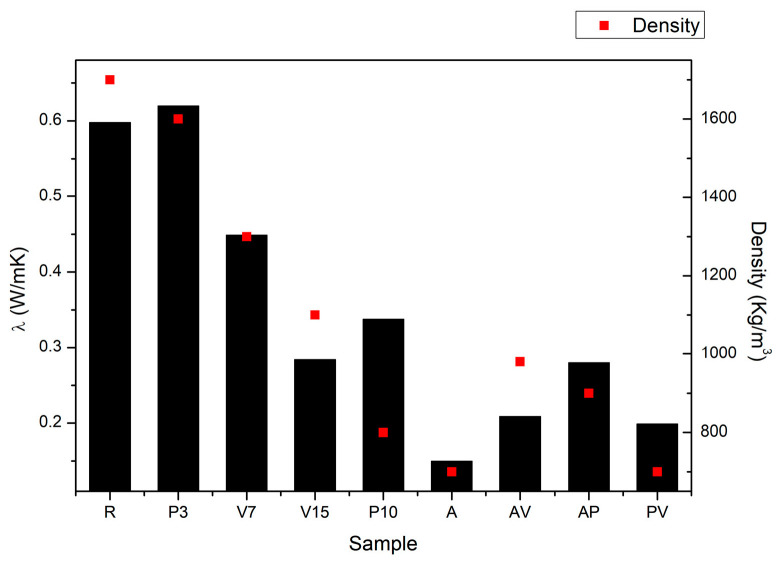
Thermal conductivity (bar, left axis) and density (square, right axis) for every sample.

Figure 6 shows values of thermal conductivity and density for each sample after 28 days of curing. In this graph, it is reasonable to expect that, with higher density, thermal conductivity is also higher. In general, it could be established that samples that contained perlite exhibited higher thermal conductivity than expected, attributed to their density values. In the case of samples that contained vermiculite, the expected behaviour was observed; the thermal conductivity decreased with the content of vermiculite and with the decrease of the mortar density. The aerogel had the lowest thermal conductivity value in line with its lowest density values (Figure 6).

On the other hand, if mortars that contain a mixture of lightweight aggregates are analysed, a combination of the behaviour of both lightweight aggregates is observed (Figure 6). Thus, the mixture that contained aerogel, when combined with vermiculite, decreased the thermal conductivity of the sample compared to the mortar incorporating vermiculite alone, independently of the vermiculite content. However, if the sample contained perlite, the improvement provided by the aerogel was insufficient to reach the expected thermal conductivity value based on its density. In the mixture that contained perlite and vermiculite, even though the density of the mortar was very low, with similar values to the sample with aerogel A, the thermal conductivity was higher than expected for these low-density values. This may be due to a predominant effect of perlite vermiculite, giving the mixture a higher thermal conductivity than expected. 

### 3.3. Thermal Performance of Mortar under Test Simulation

As explained in Section 2, to understand the response of mortars with different lightweight aggregates when exposed to heat or cold environmental conditions, a homemade testing system was designed. This system allows fixing the temperature on one side and monitoring the material’s response over time.

#### 3.3.1. Hot Conditions

**Figure 7:**
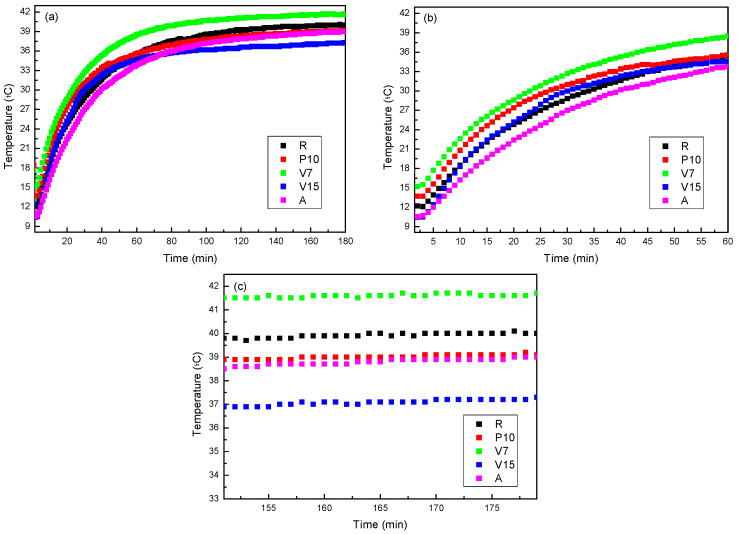
Comparison of behaviour between samples under heat conditions (showing the side not exposed to the heat source): (**a**) complete record, (**b**) heating stage, and (**c**) steady state.

The data collected from the solar simulator during a test exposing mortars to heat conditions (with a constant temperature of 40 °C on the side exposed to the heat source) are presented in Figure 7. In Figure 7a, the entire experiment is depicted, from the beginning of the heating process to the time of reaching a steady state. In this graph, it is observed that all analyzed mortars followed a standard pattern during the heating process. At the beginning of the experiment, the unexposed side showed a temperature between 10 and 15 °C. Upon turning on the lamp, the exposed side quickly reached 40 °C, while the temperature on the unexposed side increased until it stabilized, indicating the attainment of a steady state.

**Figure 8:**
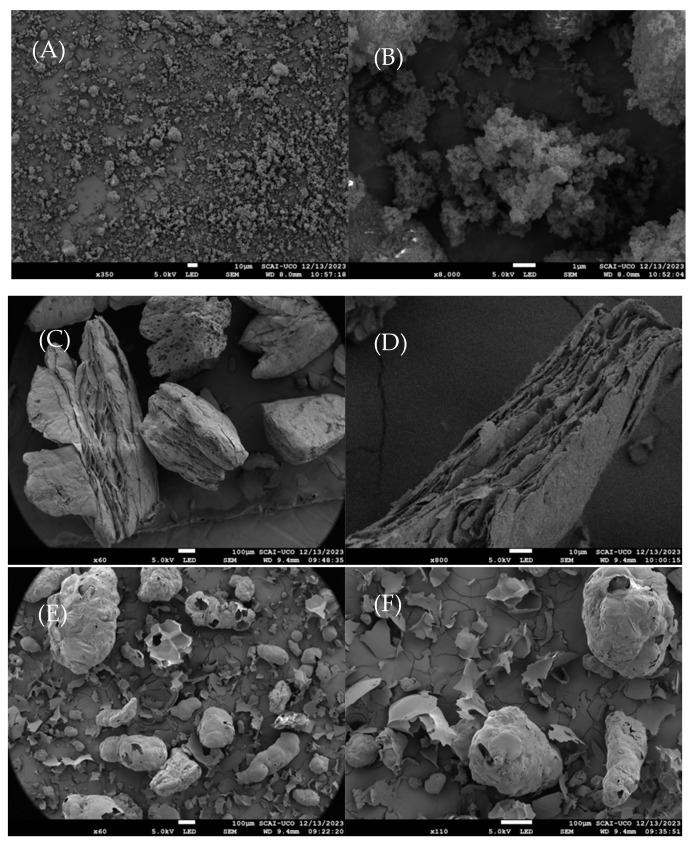
Morphology of the particles: (**A**,**B**) aerogel, (**C**,**D**) vermiculite, (**E**,**F**) perlite.

In Figure 7b, the initial heating phase for mortars with different additions is observed. Here, it can be seen that the heating curve profiles were similar for all samples, with slight deviations. These data suggest that sample A delayed the temperature increase the most, followed by samples R and V15, which practically overlapped. In contrast, sample V7 exhibited less favourable behaviour. The good performance of sample A may be attributed to the morphology of the aerogel, as its micrometric sphere structure achieved greater dispersion in the mortar, whereas the other additives consisted of larger particles with a heterogeneous morphology (see Figure 8).

The unexposed face in all mortars reached approximately 80% of its steady-state temperature after 30 min, with the exception of sample A, which contained aerogel, delaying the attainment of this value until 45 min. Based on this information, it could be deduced that the mortar that contained aerogel in its formulation was the one that transmitted heat most slowly through the material.

**Figure 9:**
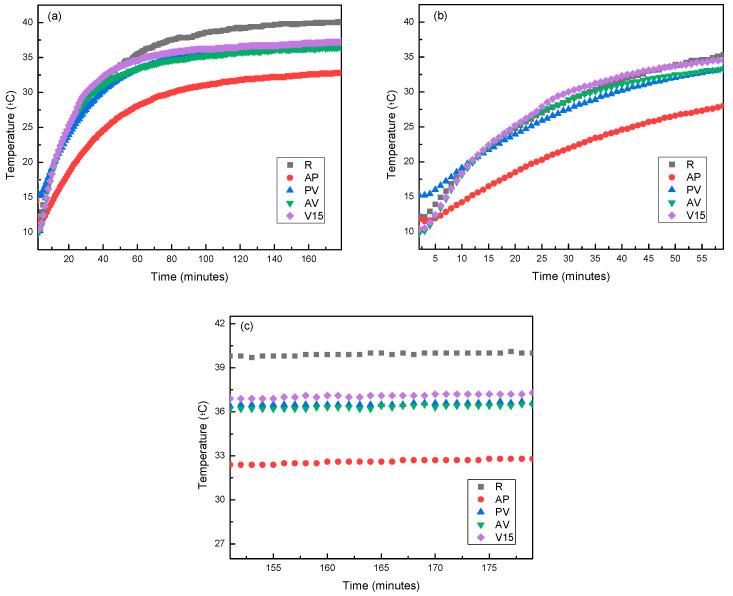
Comparison of behaviour between mixed formulations in hot conditions (showing the side not exposed to the heat source): (**a**) complete record, (**b**) heating stage, and (**c**) steady state.

If the behaviour of the samples when reaching the steady state is analysed (Figure 7c), it is possible to verify that, similarly to the non-steady state period, sample V7 was still the one exhibiting the less favourable behaviour, even behaving similarly to the reference sample as its temperature reached 40 °C, the same temperature as the side exposed to the heat source. In contrast, during the steady-state period, the sample showing the best performance was V15 instead of sample A. This shift in behaviour between these two samples can be explained based on the morphology of expanded vermiculite, which was the lightweight aggregate in this V15 sample. As observed in Figure 9, expanded vermiculite had numerous overlapping layers, enhancing its insulation level compared to aerogel, in which the air chambers formed by the spheres were clustered but not overlapped, resulting in lower thermal insulation.

A similar study was conducted with mortar formulations that use blends of lightweight aggregates (samples AP, AV, and PV). In this case (Figure 9), it could be observed that the shape of the curves was similar to that described in the previous case, so the response to the heat source was similar. However, in this instance, it was evident that mortar samples with blends of lightweight aggregates exhibited a better heat response than the reference sample, and even surpassed the performance of sample V15, which provided the best response in the previous case (Figure 9a).

If the heating ramp of the mortars is analysed in more detail, it can be observed that sample AP exhibited the best performance and was significantly different from the rest of the samples. The slope of this curve was considerably lower than that of the others, indicating that the final temperature reached will be lower compared to the other samples.

The rest of the mixtures showed a slope similar to the slope of the reference mortar. However, at the end of the heating stage, at the steady state region, a better performance for the mixtures was already observed compared to the reference case.

In the best-case scenario, mixture AP reached 80% of its final temperature on the unexposed face after 48 min, (see Table 7) a value very similar to that achieved in the case of mortars that contained only aerogel as lightweight aggregate. This indicated that mortars with a mixture of lightweight aggregates generally improved their thermal inertia compared to samples that contained only one type of lightweight aggregate.

In Figure 9c, the behaviour of the samples when reaching the steady state is depicted. Two noteworthy observations arose: overall, the performance of samples that contained blends of lightweight aggregates was superior to those without mixtures, and the best performance was observed in the sample that contained a blend of aerogel and perlite. The improved performance of this blend could be explained by a higher degree of particle dispersion in the mixture. As observed in Figure 8, aerogel particles are smaller and can integrate into the open structures of perlite, resulting in a blend that achieves thermal insulation like that of vermiculite. Another notable aspect is that, in this blend, the added lightweight aggregate constituted 20% (10% perlite and 10% aerogel), while in the other two cases, the added lightweight aggregate was 25% (15% vermiculite and 10% aerogel), indicating that the percentage of added lightweight aggregate was not the determining factor in the behaviour of the mixtures as thermal insulation.

In the last column of Table 7, the temperature difference data between the different proposed mortars and the reference are recorded, which showed up to a 2.9 °C difference in the best-case scenario for mortars that contained a single lightweight aggregate and up to a 7.2 °C difference for mortars with a mixture of lightweight aggregates in their composition.

#### 3.3.2. Cold Conditions

In the case of cold conditions, the test was conducted only for mortar samples that contained blends of lightweight aggregates, as they exhibited better performance.

**Figure 10:**
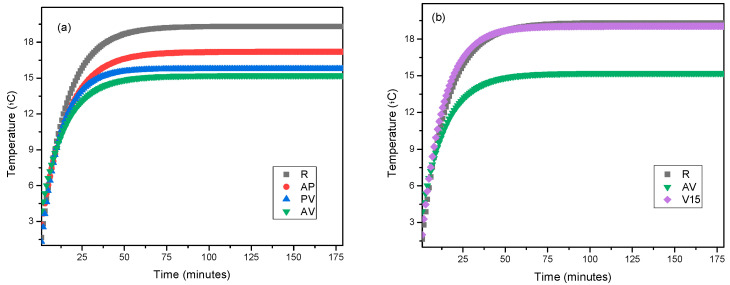
Comparison of behaviour between mixed formulations in cold conditions: (**a**) mortars with blends of lightweight aggregates, and (**b**) mortar with 15% vermiculite.

In Figure 10, the behaviour of the samples under cold conditions is presented. During this test, the samples were heated with an infrared lamp, and the temperature on the external surface of the sample (exposed to 4 °C) was monitored to evaluate the heat losses through the mortar sample. In this case, it could be observed that, in all the mixtures, the starting temperature of the experiment was 4 °C, which was the temperature of the room where the measurement equipment was located. Focusing on the mixtures, it could be observed that the behaviour of the samples varied depending on the combination of the lightweight aggregates used (Figure 10a), where it seemed that the samples that contained vermiculite as a lightweight aggregate exhibited better performance, regardless of the other lightweight aggregate that was part of the mixture. This suggests that vermiculite is the determining factor for achieving the highest thermal insulation in the samples.

To determine whether vermiculite is indeed the determining factor for good performance against cold conditions in mortars, the test was also conducted on the sample that contained only vermiculite at 15% as a lightweight aggregate (Figure 10b). It was confirmed that when vermiculite acts alone, the behaviour was like that of the reference sample, meaning the incorporation of 15% vermiculite into the mortar did not influence the response to cold conditions. These results highlight that, in the case of the response to cold conditions, the percentage of lightweight aggregate added to the mortar was the determining factor for achieving the best performance, rather than the type of lightweight aggregate added.

As indicated in Table 8, in this case, the samples that took the longest to reach 80% of their insulation level were the PV and AV samples, highlighting once again that the mixtures that contained a greater amount of lightweight aggregate were the ones providing a better response to cold conditions. In this instance, the temperature difference between the different mortar mixtures and the reference is recorded in the last column of Table 8, where it was observed that, for the AV sample, the difference reached compared to the reference mortar was 4.4 °C.

## 4. Conclusions

This article focused on the study of incorporating lightweight aggregates (aerogel, perlite, and vermiculite), or mixtures thereof, into a cement-based mortar to enhance its thermal insulation properties.

The incorporation of lightweight aggregates improved the thermal insulation of the mortar. In general, as the content of lightweight aggregate increased, the thermal conductivity decreased. However, the sample that contained aerogel exhibited the best performance, improving the thermal conductivity value by almost 75%.

The design of a solar simulation system under cold and hot conditions allowed us to assess the material’s performance under simulated real conditions.

The response of these mortars to heat indicated that when using a unique type of lightweight aggregate, aerogel exhibited the highest thermal inertia, while the best insulation performance was shown by the sample with vermiculite, with a 15% improvement. When using mixtures of lightweight aggregates, the sample that contained 10% perlite and 10% aerogel demonstrated the best performance in both thermal inertia and final insulation capacity.

In the case of using a single lightweight aggregate, the insulation capacity reached up to 3 °C, and in the case of mixtures of lightweight aggregates, the improvement extended to 7.2 °C.

Under cold conditions, the response of the mortars was different. In this case, the samples with the highest content of lightweight aggregate in their composition exhibited the best performance, achieving an improvement of up to 4.5 °C compared to the reference mortar.

Lightweight aggregates led to a decrease in mechanical resistances; however, in all cases the minimum resistance to be considered a plaster mortar was fulfilled. A direct correlation was found between the amount of water used and compressive strength; increasing the amount of water decreases the compressive strength of the mortar. An exception was observed in the case of the mortar that contained aerogel which exhibited better behaviour than the expected behaviour. The worst behaviour was observed for the mortar with a high percentage of vermiculite.

## Figures and Tables

**Table 1:** Summary of the mixture proportions (by weight %) of the studied mortars.

Raw Material/%	R	P3	P10	V7	V15	A	AP	AV	PV
Cement	22	22	22	22	22	22	22	22	22
Dolomitic sand	71.6	68.6	61.6	64.6	56.6	61.6	51.6	46.6	46.6
Filler	5.4	5.4	5.4	5.4	5.4	5.4	5.4	5.4	5.4
Fibre	0.7	0.7	0.7	0.7	0.7	0.7	0.7	0.7	0.7
Additives	0.3	0.3	0.3	0.3	0.3	0.3	0.3	0.3	0.3
Perlite		3	10				10		10
Vermiculite				7	15			15	15
Aerogel						10	10	10	

**Table 2:** Thermal conductivity values for raw materials.

Raw Material	Thermal Conductivity (λ = W/m·K)	λ Standard Deviation
Cement	0.116	0.001
Dolomite	0.444	0.007
Filler	0.13	0.02
Fibre	0.075	0.005
Water retainer	0.108	0.003
Waterproofing	0.119	0.002
Perlite	0.05	0.02
Vermiculite	0.077	0.005
Aerogel	0.075	0.004

**Table 3:** Density and consistency of the studied mortars.

Mix Code	Powder Density (Kg/m^3^)	Fresh Density (Kg/m^3^)	Mixing Water (%)	Consistency (mm)
R	1985	2420	19.9	170
P3	1845	2250	20.4	175
P10	720	1350	54.6	175
V7	1420	1500	33.1	167
V15	1000	1310	45.2	170
A	1050	1200	29.0	160
AP	590	1425	57.2	180
AV	800	1200	70.4	175
PV	610	1230	75.9	180

**Table 4:** Flexural and compressive strength at an early age (7 days curing).

Mix Code	Flexural Strength (N/mm^2^)	% Loss Relative to the Reference Mix	Compressive Strength (N/mm^2^)	% Loss Relative to the Reference Mix
R	3.5	--	10.3	--
P3	3.3	5.7	9.7	5.8
P10	1.0	71.4	2.1	79.6
V7	1.4	60	3.3	67.9
V15	0.6	82.9	0.7	93.2
A	2.3	34.3	4.2	59.2
AP	0.7	80	1.3	87.4
AV	0.3	91.4	0.5	95.2
PV	0.4	88.6	0.7	93.2

**Table 5:** Properties of mortar in the hardened state.

Mix Code	Density at 28 d (Kg/m^3^)	Flexural Strength (N/mm^2^)	% Loss Relative to the Reference Mix	Compressive Strength (N/mm^2^)	% Loss Relative to the Reference Mix
R	1700	5.4	--	15.4	--
P3	1600	5.6	0	14.2	7.8
P10	1100	1.3	76.4	3.4	77.9
V7	1300	2.5	54.6	5.4	64.9
V15	900	1.0	81.8	2.0	87.1
A	800	2.4	56.4	4.4	71.4
AP	980	0.8	85.5	1.9	87.7
AV	700	0.4	92.7	0.8	94.8
PV	700	0.5	90.9	0.9	94.2

**Table 6:** Thermal conductivity values for different mixes evaluated with portable devices.

Mix Code	Thermal Conductivity (λ = W/m·K)	Improvement in λ (%)	λ Standard Deviation
Reference	0.598	--	0.006
P3	0.62	--	0.02
P10	0.338	43.5	0.005
V7	0.449	24.9	0.005
V15	0.284	52.5	0.006
A	0.15	74.9	0.01
AP	0.28	53.2	0.01
AV	0.209	67.0	0.009
PV	0.199	66.7	0.004

**Table 7:** Temperature data for the different samples.

Mix Code	Maximum Temperature, Unexposed Face, Steady State (°C)	80%	Time (Minutes)	∆T
Reference	39.9	31.9	42	0
P10	39	31.2	32	0.9
V7	41.0	33.3	32	0
V15	37	29.6	30	2.9
A	38.8	31	45	1.1
AP	32.7	26.1	48	7.2
PV	36.5	29.2	36	3.4
AV	36.3	29	31	3.6

**Table 8:** Temperature data for the different samples.

Mix Code	Maximum Temperature, Unexposed Face, Steady State (°C)	80%	Time (Minutes)	∆T
Reference	19.4	15.5	25.1	0
AP	17.3	13.8	25.8	2.1
PV	15.8	12.7	20.5	3.6
AV	15	12	19.8	4.4

## Data Availability

Data are contained within the article.
